# Bilateral Double Ureters with Bladder Neck Diverticulum in a Nigerian Woman Masquerading as an Obstetric Fistula

**DOI:** 10.1155/2014/801063

**Published:** 2014-12-23

**Authors:** Imran O. Morhason-Bello, Sikiru A. Adebayo, Rukiyat A. Abdusalam, Oluwasomidoyin O. Bello, Kehinde H. Odubamowo, Olatunji O. Lawal, E. Oluwabunmi Olapade-Olaopa, Oladosu A. Ojengbede

**Affiliations:** ^1^Genitourinary Medicine and Urogynaecology Unit, Department of Obstetrics and Gynaecology, Faculty of Clinical Sciences, College of Medicine, University of Ibadan/University College Hospital, Ibadan, Oyo State, Nigeria; ^2^Urology Division, Department of Surgery, Faculty of Clinical Sciences, College of Medicine, University of Ibadan/University College Hospital, Ibadan, Oyo State, Nigeria; ^3^PIUTA Ibadan Centre, University College Hospital, Ibadan, Nigeria

## Abstract

A 43-year-old woman presented with 20-year history of leakage of urine per vaginam. She had one failed repair attempt. Pelvic examination with dye test showed leakage of clear urine suggestive of ureterovaginal fistula. The preoperative intravenous urogram revealed duplex ureter and cystoscopy showed normally cited ureteric orifices with two other ectopic ureteric openings and bladder diverticula. The definitive surgery performed was ureteric reimplantation (ureteroneocystostomy) of the two distal ureteric to 2 cm superiolateral to the two normal orifices and diverticuloplasty. There was resolution of urinary incontinence after surgery. Three months after surgery, she had urodynamic testing done (cystometry), which showed 220 mLs with no signs of instability or leakage during filling phase but leaked on coughing at maximal bladder capacity. This is to showcase some diagnostic dilemma that could arise with obstetric fistula, which is generally diagnosed by clinical assessment.

## 1. Introduction

Urinary incontinence remains a psychologically distressing morbidity to women, and one of the commonest causes after childbirth in developing countries is obstetric fistula (OF) [1, 2]. At the moment, Nigeria has the largest burden of OF. According to the 2008 National Demographic Health Survey, the estimated prevalence of OF is about 0.4 percent. This figure is however believed to have underestimated the current reality in the country judging by the volume of backlog in the various communities and recent upsurge in other areas. The diagnosis of OF is mostly clinical but in rare occasions detailed urological evaluation might be necessary to determine the cause and also to exclude differential diagnosis [3, 4]. Some causes of urinary incontinence that could mimic OF include nonfistulous incontinence (stress, urge, and overflow), urinary tract infection, and congenital malformation of urological system such as ectopically inserted duplicated ureter, bladder diverticulum, and ureterocele [4, 5]. Evaluation of some of these conditions might require imaging (ultrasonography, intravenous urography, micturating, and cystourethrogram), cystoscopy, and urodynamic studies, which are not readily available in most fistula-designated sites in developing countries.

In general, congenital duplication of ureter is an uncommon presentation of urinary incontinence in the adult population [6]. In most cases, it manifests during childhood and may be associated with other renal abnormalities such as duplex kidney, bladder diverticulum, and duplication [7, 8]. Duplex ureter is a product of two different ureteric buds that originate from a single Wolffian duct or a single bud splits into two ureters on each kidney at the 4th week of embryonic development. This abnormality is mostly unilateral in nature [6]. The duplication could be partial (bifid) in which two ipsilateral ureters drain into the bladder via a common trunk or it may be complete where both ureters drain separately either into the bladder or into ectopic sites such as vagina, urethra, or vulva vestibule [7]. According to Weigert-Meyer rule, the upper pole of the kidney is commonly drained by the ureter that is below and medial in about 90 percent of cases [9]. Women with duplex ureters are mostly asymptomatic and they are usually detected accidentally during other investigations. Some are however discovered during evaluation for recurrent urinary tract infection, vesicoureteral reflux, and urinary incontinence [9].

This case is presented to showcase the importance of thorough preoperative evaluation of a woman with urinary incontinence following childbirth from a rare cause, which was initially thought to be OF, and the need to advocate for modern tools to support care in resource limited setting.

## 2. Case Report

A 43-year-old Nigerian Yoruba woman para 1 (not alive) presented at the Urogynaecology Clinic of the University College Hospital, Ibadan, on August 7, 2012, with 20-year history of leakage of urine per vaginam, which started 2 weeks after spontaneous vaginal delivery at term at a public secondary health facility. There was no history of augmentation, instrumentation, or difficult delivery. The labour however lasted 24 hours. She delivered a stillborn baby but could not remember the residual birth weight. She has urge to void despite the urinary leakage, and there was no stress incontinence. There were no urinary symptoms suggestive of infection. Initially, the patient was conservatively treated with urethral catheterization for continuous bladder drainage of urine for 6 weeks on outpatient basis with no resolution of symptoms after its removal. She had prophylactic antibiotic for 2 weeks during catheterization period. She had a failed vaginal surgical repair 19 years ago at a fistula center and had been living with the leakage of urine till presentation in our facility. She does not have any other medical condition. She menstruates regularly prior to surgery.

The patient separated from her husband shortly after the onset of urinary leakage, and she has been living alone with no family support. To avoid embarrassment of stigma, she relocated thrice within Nigeria and to neighboring Republic of Benin.

The patient is a middle-aged woman who is 1.7 m tall and weighs 82.0 kilograms. Physical and other systems examinations were normal except pelvic region. Bimanual digital vaginal examination in dorsal position shows reduced vagina length of 5 cm with obliteration of the fornices. There was no palpable defect on the anterior and posterior vaginal walls. The uterus was of normal size and adnexae were free. Examination under anaesthesia and dye test showed leakage of clear urine that was not dye-stained in the vagina after instillation of 100 mLs of methylene-blue dye into the urinary bladder. A diagnosis of ureterovaginal fistula was made.

**Figure 1:**
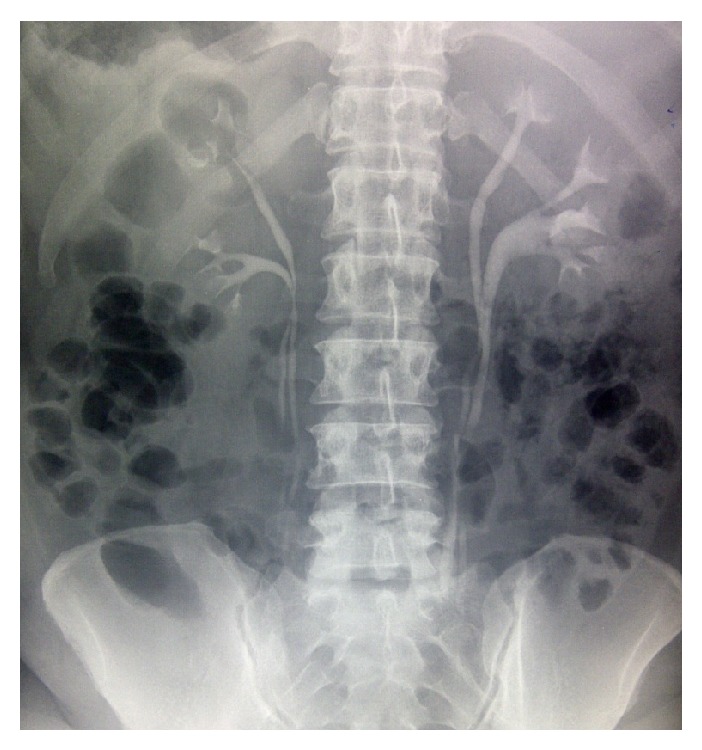
Intravenous urography showing the bilateral double ureters draining into the bladder.

**Figure 2:**
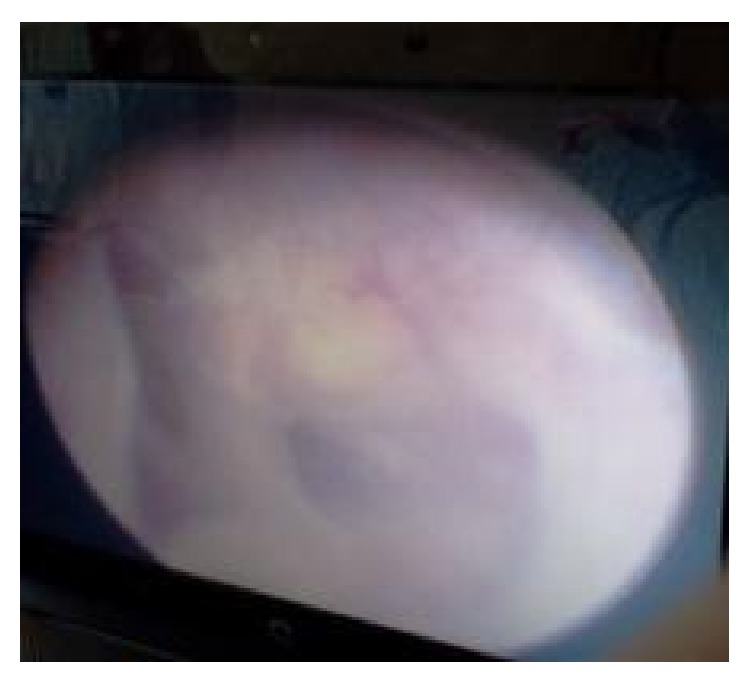
Cystoscopy result showing diverticulum near the bladder neck.

Preoperative investigations done are as follows: haematocrit was 36 percent; full blood count, electrolyte, urea, and creatinine were normal. Urinalysis showed high level of nitrite and proteinuria. Microscopy, culture, and sensitivity of urine sample yielded a significant growth of* Escherichia coli* sensitive to nitrofurantoin and cefuroxime. Intravenous urography revealed prompt excretion of the contrast bilaterally. Both kidneys have duplication of collecting system with duplex ureters draining into the bladder. Opacification of urinary bladder shows a fairly smooth with a double density shadow and significant contrast below it suggesting vesicovaginal fistula (Figure 1). Cystoscopy showed normal urethral caliber, and there was a bladder diverticulum with an opening inferior to the trigone, multiple ureteric orifices—two were located within the trigone while the other two were distal (1 to 2 cm) to the bladder neck. There was also a visible 8 bladder diverticulum measuring 2 cm by 3 cm inferior to the trigone (the lower edge was about 1 cm to the bladder neck) (Figure 2). The conclusion at cystoscopy was bladder diverticulum and ectopic ureteric openings.

The patient had laparotomy done with combined spinal epidural regional anaesthesia. The intraoperative findings include moderate pelvic adhesions and bulky uterus with visualized normal ovaries and tubes. There were bilateral double ureters that drain separately into the bladder. Both kidneys were normal in size and location. Four ureteric orifices were visualized at the same location as described during cystoscopy. There was a bladder diverticulum measuring 2 cm by 3 cm located at about 1 cm short of bladder neck. The definitive surgery performed was ureteric reimplantation (ureteroneocystostomy) of the two distal ureteric openings to 2 cm superiolateral points of the two normally sited ureteric orifices, and diverticuloplasty. Ureteric stents were used to cannulate the neoureteric openings and a suprapubic cystostomy and urethral were catheterized each with size of 16 G Foley catheter. The woman had perioperative broad-spectrum antibiotics and opioid analgesics and hydrated with 4 liters of intravenous fluids in the first 24 hours with satisfactory urinary output. Thereafter, she was commenced on oral fluids and diets with progressive improvement of her clinical status. The ureteric, suprapubic, and urethral catheters were removed on days 10, 14, and 21, respectively, and the patient had uneventful postoperative experience. She was discharged home after satisfactory bladder training on the 22nd postoperative day. She had urgency, which was managed with anticholinergic drug with resolution of symptoms. She resumed her menstrual period without hindrance.

**Figure 3:**
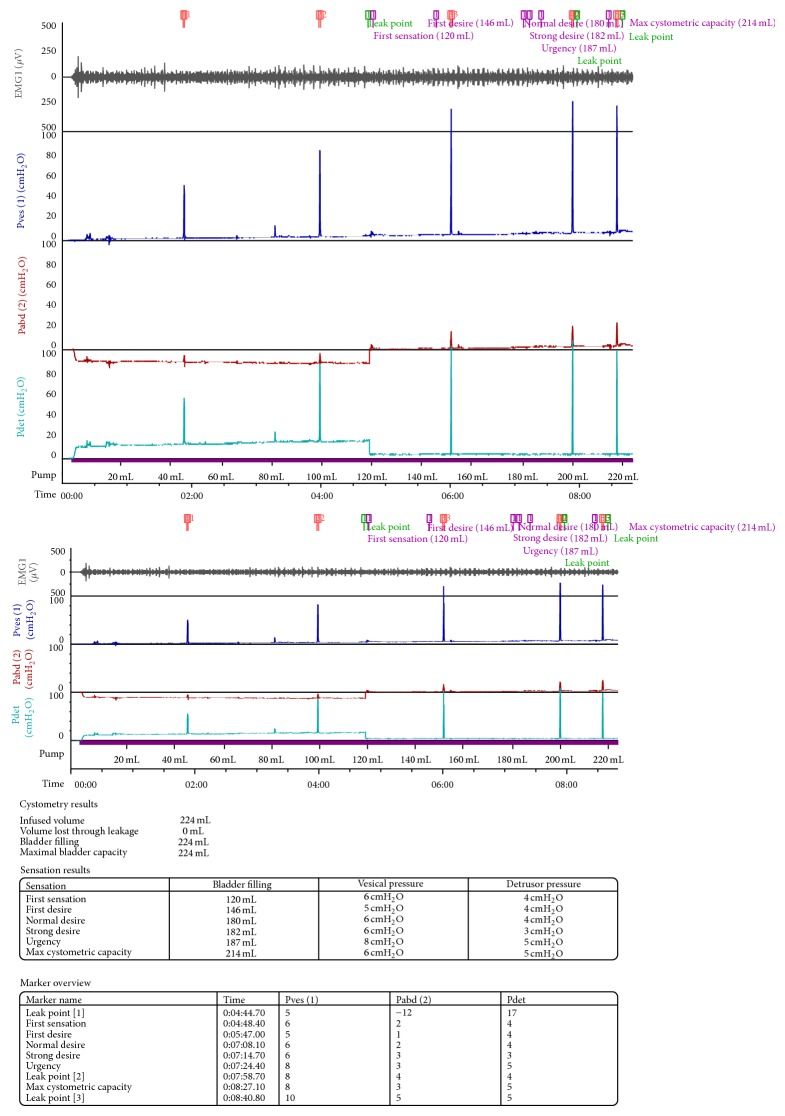
Postoperative cystometry.

Three months after the surgery, she had urodynamic studies performed at the PIUTA Ibadan Center at the Surgical Outpatient Department of the hospital with Solar Blue MMS Machine, Netherlands. The study showed a reduced bladder capacity of approximately 220 mLs with no signs of instability or leakage during filling phase but leaked on coughing at maximal bladder capacity (Figure 3). She voided normally and to completion at the end of the study. She had no fresh complaint at subsequent clinics and was discharged from the follow-up clinic. During the discharge discussion, she expressed desire to have children in the future, and she was counseled on hospital supervised delivery with a specialist.

## 3. Discussion

Duplex ureter with ectopic orifice is associated with urinary incontinence and it often presents early in life where corrective surgery is usually offered [9, 10]. The majority of those presenting with incontinence often have ectopic orifice that is extravesical in location [9]. This abnormality is commonly seen in females [9].

The patient was initially thought to have OF due to prolonged labour by the team of physicians who managed her urinary leakage after childbirth and this is why 6-week catheterization was employed in line with the conservative management protocol. In addition, the failed attempt at vaginal repair when no defect exists is difficult to explain in this patient. Again, this is another erroneous manifestation that postpartum urinary incontinence is due to OF in this environment. These two incidents bring to attention the need for a thorough evaluation of patients when they present with their clinical condition before embarking on definitive care to avoid undue risk and unnecessary wastage of meager resources. The absence of urine stained with dye during the examination under anaesthesia rules out the possibility of vesicovaginal fistula but suggests the possibility of ureteric fistula. The failure of intravenous urography to show any evidence of urine extravasation coupled with the preoperative cystoscopy that revealed urine ejection from the four ureteric orifices within the bladder rules out ureteric fistula.

The presentation of urine leakage after childbirth in this type of patient could be explained by any of the following possibilities. First, it is plausible that the prolonged obstructed labour might have distorted the bladder and this could reduce the intraurethral pressure relative to the intravesical pressure coupled with the abnormally located ureteric orifices [11]. Second, the presence of bladder diverticulum next to the bladder neck might also be responsible for the incontinence complaint [12]. Thirdly, the bladder irritation from recurrent urinary infections might be responsible. However, it is unlikely to be the sole cause because the urine leakage persisted after treatment of the infection.

The two-decade duration of clinical presentation resolved after reimplantation of the two abnormally located ureteric orifices and closure of the diverticulum. The postoperative urodynamic evaluation revealed reduced bladder capacity and leakage at maximal bladder capacity. The literature is replete with the observation that reduced bladder capacity is a common complication of diverticuloplasty and the leakage observed at urodynamic study was not symptomatic because the woman had adjusted to the bladder capacity by frequent voiding. The patient coped with satisfaction to the voiding pattern and frequency. She was thereafter counseled and followed up in the Urogynaecology Clinic.

In conclusion, this case demonstrates a rare differential of OF which was unraveled by a painstaking evaluation process that is necessitated out of the routine examination. This woman could have been referred early to a health facility that has capacity to investigate and manage her clinical condition rather than employing a “trial and error” gaffe.
